# (*E*)-5-(2-Nitro­prop-1-en­yl)-2,3-dihydro-1-benzofuran

**DOI:** 10.1107/S1600536809023277

**Published:** 2009-06-27

**Authors:** Hong Xu, Hongshun Sun, Ning Xu

**Affiliations:** a Department of Chemical Engineering, Nanjing College of Chemical Technology, Geguan Road No. 265 Nanjing, Nanjing 210048, People’s Republic of China; b Department of Applied Chemistry, Nanjing College of Chemical Technology, Geguan Road No. 265 Nanjing, Nanjing 210048, People’s Republic of China

## Abstract

The asymmetric unit of the title compound, C_11_H_11_NO_3_, contains two crystallographically independent mol­ecules. The aromatic rings are oriented at a dihedral angle of 56.17 (5)°. The furan rings adopt envelope conformations. Intra­molecular C—H⋯N inter­actions results in the formation of two six-membered rings with twisted conformations. In the crystal structure, three weak C—H⋯π inter­actions are found.

## Related literature

For bond-length data, see: Allen *et al.* (1987 ▶).
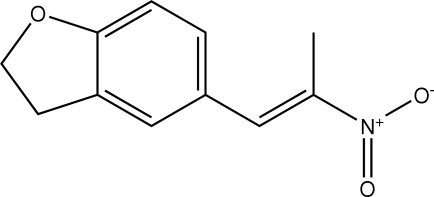


## Experimental

### 

#### Crystal data

C_11_H_11_NO_3_
*M* *_r_* = 205.21
Monoclinic, 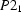

*a* = 6.1280 (12) Å
*b* = 15.369 (3) Å
*c* = 11.193 (2) Å
β = 101.38 (3)°
*V* = 1033.5 (4) Å^3^
*Z* = 4
Mo *K*α radiation
μ = 0.10 mm^−1^
*T* = 294 K
0.20 × 0.10 × 0.10 mm

#### Data collection

Enraf–Nonius CAD-4 diffractometer
Absorption correction: ψ scan (North *et al.*, 1968 ▶) *T*_min_ = 0.981, *T*_max_ = 0.990
2548 measured reflections
2335 independent reflections
1289 reflections with *I* > 2σ(*I*)
*R*_int_ = 0.031
3 standard reflections frequency: 120 min intensity decay: 1%

#### Refinement

*R*[*F* ^2^ > 2σ(*F* ^2^)] = 0.055
*wR*(*F* ^2^) = 0.163
*S* = 1.01
2335 reflections
271 parameters
H-atom parameters constrained
Δρ_max_ = 0.16 e Å^−3^
Δρ_min_ = −0.14 e Å^−3^

### 

Data collection: *CAD-4 Software* (Enraf–Nonius, 1989 ▶); cell refinement: *CAD-4 Software*; data reduction: *XCAD4* (Harms & Wocadlo, 1995 ▶); program(s) used to solve structure: *SHELXS97* (Sheldrick, 2008 ▶); program(s) used to refine structure: *SHELXL97* (Sheldrick, 2008 ▶); molecular graphics: *ORTEP-3 for Windows* (Farrugia, 1997 ▶) and *PLATON* (Spek, 2009 ▶); software used to prepare material for publication: *SHELXL97* and *PLATON*.

## Supplementary Material

Crystal structure: contains datablocks global, I. DOI: 10.1107/S1600536809023277/hk2683sup1.cif

Structure factors: contains datablocks I. DOI: 10.1107/S1600536809023277/hk2683Isup2.hkl

Additional supplementary materials:  crystallographic information; 3D view; checkCIF report

## Figures and Tables

**Table 1:** Hydrogen-bond geometry (Å, °)

*D*—H⋯*A*	*D*—H	H⋯*A*	*D*⋯*A*	*D*—H⋯*A*
C7—H7*A*⋯N1	0.93	2.58	3.096 (8)	116
C18—H18*A*⋯N2	0.93	2.60	3.094 (7)	114
C4—H4*A*⋯*Cg*4	0.97	2.88	3.673 (8)	140
C8—H8*A*⋯*Cg*3^i^	0.93	2.92	3.578 (8)	129
C15—H15*B*⋯*Cg*2	0.97	2.85	3.636 (7)	139
C19—H19*A*⋯*Cg*2^ii^	0.93	2.75	3.497 (7)	139

Symmetry codes: (i) 
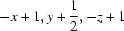
; (ii) 
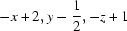
. *Cg*2, *Cg*3 and *Cg*4 are the centroids of the C2/C3/C5–C8, O4/C12–C15 and C13/C14/C16–C19 rings, respectively.

**Fig. 1.**
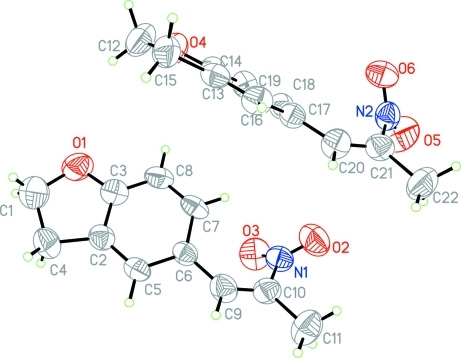
The molecular structure of the title molecule, with the atom-numbering scheme. Displacement ellipsoids are drawn at the 30% probability level.

## supplementary crystallographic information

### Comment

Some derivatives of benzol are important chemical materials. We report herein the crystal structure of the title compound.

The asymmetric unit of the title compound contains two crystallographically independent molecules (Fig. 1), in which the bond lengths (Allen *et al.*, 1987) and angles are within normal ranges. Rings B (C2/C3/C5-C8) and D (C13/C14/C16-C19) are, of course, planar and the dihedral angle between them is B/D = 56.17 (5)°. Rings A (O1/C1-C4) and C (O4/C12-C15) adopt envelope conformations with atoms C1 and C12 displaced by -0.189 (4) and -0.124 (5) Å from the planes of the other rings atoms. The moieties E (N1/C9-C11) and F (N2/C20-C22) are planar [the maximum deviations are -0.002 (4) and 0.016 (4) Å for atoms C10 and C21, respectively], and they are oriented with respect to the adjacent rings at dihedral angles of B/E = 32.39 (4) and D/F = 35.26 (5) °. Intramolecular C-H···N interactions (Table 1) results in the formations of two six-membered rings (N1/C6/C7/C9/C10/H7A) and (N2/C17/C18/C20/C21/H18A) having twisted conformations.

In the crystal structure, three weak C—H···π interactions are found.

### Experimental

For the preparation of the title compound, ammonium acetate (0.92 g, 12 mmol) was added to a solution of 5-formyl-2,3-dihydrobenzofuran (3.3 g, 22.3 mmol) in nitroethane (10 ml). The mixture was heated with stirring to 383 K in an oil bath for 3.5 h. The volatiles were then removed by rotary evaporation. The crude product was triturated in cold CH_3_OH (10 ml), collected by filtration (yield; 3.06 g, 67%). Crystals suitable for X-ray analysis were obtained by slow evaporation of a methanol solution.

### Refinement

H atoms were positioned geometrically, with C-H = 0.93, 0.97 and 0.96 Å for aromatic, methylene and methyl H, respectively, and constrained to ride on their parent atoms, with U_iso_(H) = xU_eq_(C), where x = 1.5 for methyl H and x = 1.2 for all other H atoms. In the absence of significant anomalous dispersion effects, Friedel pairs were averaged.

### Crystal data

**Table:**

C_11_H_11_NO_3_	*F*(000) = 432
*M**_r_* = 205.21	*D*_x_ = 1.319 Mg m^−^^3^
Monoclinic, *P*2_1_	Mo *K*α radiation, λ = 0.71073 Å
Hall symbol: P 2yb	Cell parameters from 25 reflections
*a* = 6.1280 (12) Å	θ = 10–13°
*b* = 15.369 (3) Å	µ = 0.10 mm^−^^1^
*c* = 11.193 (2) Å	*T* = 294 K
β = 101.38 (3)°	Block, colorless
*V* = 1033.5 (4) Å^3^	0.20 × 0.10 × 0.10 mm
*Z* = 4	

### Data collection

**Table:**

Enraf–Nonius CAD-4 diffractometer	1289 reflections with *I* > 2σ(*I*)
Radiation source: fine-focus sealed tube	*R*_int_ = 0.031
graphite	θ_max_ = 27.0°, θ_min_ = 1.9°
ω/2θ scans	*h* = 0→7
Absorption correction: ψ scan (North *et al.*, 1968)	*k* = 0→19
*T*_min_ = 0.981, *T*_max_ = 0.990	*l* = −14→14
2548 measured reflections	3 standard reflections every 120 min
2335 independent reflections	intensity decay: 1%

### Refinement

**Table:**

Refinement on *F*^2^	Secondary atom site location: difference Fourier map
Least-squares matrix: full	Hydrogen site location: inferred from neighbouring sites
*R*[*F*^2^ > 2σ(*F*^2^)] = 0.055	H-atom parameters constrained
*wR*(*F*^2^) = 0.163	*w* = 1/[σ^2^(*F*_o_^2^) + (0.08*P*)^2^] where *P* = (*F*_o_^2^ + 2*F*_c_^2^)/3
*S* = 1.01	(Δ/σ)_max_ < 0.001
2335 reflections	Δρ_max_ = 0.16 e Å^−^^3^
271 parameters	Δρ_min_ = −0.14 e Å^−^^3^
Primary atom site location: structure-invariant direct methods	

### Special details

**Table:**

Geometry. All e.s.d.'s (except the e.s.d. in the dihedral angle between two l.s. planes) are estimated using the full covariance matrix. The cell e.s.d.'s are taken into account individually in the estimation of e.s.d.'s in distances, angles and torsion angles; correlations between e.s.d.'s in cell parameters are only used when they are defined by crystal symmetry. An approximate (isotropic) treatment of cell e.s.d.'s is used for estimating e.s.d.'s involving l.s. planes.
Refinement. Refinement of *F*^2^ against ALL reflections. The weighted *R*-factor *wR* and goodness of fit *S* are based on *F*^2^, conventional *R*-factors *R* are based on *F*, with *F* set to zero for negative *F*^2^. The threshold expression of *F*^2^ > σ(*F*^2^) is used only for calculating *R*-factors(gt) *etc*. and is not relevant to the choice of reflections for refinement. *R*-factors based on *F*^2^ are statistically about twice as large as those based on *F*, and *R*- factors based on ALL data will be even larger.

### Fractional atomic coordinates and isotropic or equivalent isotropic displacement parameters (Å^2^)

**Table:**

	*x*	*y*	*z*	*U*_iso_*/*U*_eq_	
O1	0.7614 (7)	0.4628 (3)	0.6830 (3)	0.0846 (13)	
O2	0.2010 (8)	0.4770 (4)	0.0356 (4)	0.1031 (16)	
O3	0.2751 (6)	0.3723 (3)	0.1606 (4)	0.0921 (14)	
O4	0.2376 (7)	0.6709 (3)	0.6129 (4)	0.0784 (14)	
O5	−0.3141 (7)	0.6604 (4)	−0.0306 (5)	0.1172 (19)	
O6	−0.2291 (7)	0.7649 (3)	0.0949 (4)	0.0878 (13)	
N1	0.3306 (7)	0.4276 (3)	0.0942 (5)	0.0659 (13)	
N2	−0.1822 (7)	0.7083 (3)	0.0289 (4)	0.0636 (12)	
C1	0.9850 (13)	0.4324 (5)	0.7308 (6)	0.098 (2)	
H1A	0.9828	0.3902	0.7950	0.118*	
H1B	1.0783	0.4808	0.7651	0.118*	
C2	0.9084 (8)	0.4194 (3)	0.5194 (5)	0.0548 (13)	
C3	0.7336 (8)	0.4598 (4)	0.5594 (5)	0.0563 (15)	
C4	1.0773 (10)	0.3913 (5)	0.6293 (5)	0.0720 (16)	
H4A	1.0837	0.3285	0.6369	0.086*	
H4B	1.2244	0.4135	0.6265	0.086*	
C5	0.9035 (8)	0.4120 (3)	0.3964 (5)	0.0540 (14)	
H5A	1.0207	0.3856	0.3687	0.065*	
C6	0.7209 (7)	0.4443 (3)	0.3137 (5)	0.0490 (12)	
C7	0.5452 (8)	0.4844 (4)	0.3565 (5)	0.0550 (14)	
H7A	0.4236	0.5060	0.3014	0.066*	
C8	0.5529 (9)	0.4917 (4)	0.4809 (5)	0.0621 (14)	
H8A	0.4370	0.5179	0.5101	0.074*	
C9	0.7270 (8)	0.4392 (4)	0.1835 (5)	0.0627 (16)	
H9A	0.8699	0.4389	0.1670	0.075*	
C10	0.5669 (9)	0.4350 (4)	0.0849 (5)	0.0624 (15)	
C11	0.5970 (10)	0.4375 (5)	−0.0440 (6)	0.084 (2)	
H11A	0.7525	0.4424	−0.0456	0.126*	
H11B	0.5188	0.4866	−0.0846	0.126*	
H11C	0.5390	0.3849	−0.0847	0.126*	
C12	0.4566 (12)	0.7054 (5)	0.6645 (5)	0.086 (2)	
H12A	0.5519	0.6592	0.7046	0.103*	
H12B	0.4449	0.7498	0.7244	0.103*	
C13	0.3874 (8)	0.7151 (4)	0.4498 (5)	0.0555 (15)	
C14	0.2113 (8)	0.6758 (4)	0.4893 (5)	0.0546 (13)	
C15	0.5551 (9)	0.7439 (4)	0.5618 (5)	0.0687 (16)	
H15A	0.7020	0.7205	0.5614	0.082*	
H15B	0.5640	0.8069	0.5674	0.082*	
C16	0.3809 (8)	0.7200 (4)	0.3276 (5)	0.0590 (14)	
H16A	0.4996	0.7453	0.3001	0.071*	
C17	0.2018 (8)	0.6882 (4)	0.2430 (5)	0.0512 (13)	
C18	0.0343 (9)	0.6479 (3)	0.2890 (5)	0.0588 (14)	
H18A	−0.0843	0.6241	0.2339	0.071*	
C19	0.0323 (9)	0.6409 (4)	0.4116 (5)	0.0617 (16)	
H19A	−0.0839	0.6141	0.4397	0.074*	
C20	0.2089 (8)	0.6926 (4)	0.1136 (5)	0.0617 (14)	
H20A	0.3522	0.6917	0.0978	0.074*	
C21	0.0500 (8)	0.6977 (4)	0.0139 (5)	0.0609 (15)	
C22	0.0741 (11)	0.6899 (6)	−0.1155 (5)	0.096 (2)	
H22A	0.2286	0.6834	−0.1187	0.144*	
H22B	0.0163	0.7413	−0.1593	0.144*	
H22C	−0.0072	0.6400	−0.1518	0.144*	

### Atomic displacement parameters (Å^2^)

**Table:**

	*U*^11^	*U*^22^	*U*^33^	*U*^12^	*U*^13^	*U*^23^
O1	0.106 (3)	0.093 (3)	0.061 (3)	0.016 (3)	0.032 (2)	0.002 (2)
O2	0.077 (3)	0.119 (4)	0.110 (4)	0.027 (3)	0.012 (3)	0.014 (3)
O3	0.070 (3)	0.107 (4)	0.104 (3)	−0.020 (3)	0.028 (2)	0.014 (3)
O4	0.090 (3)	0.088 (4)	0.057 (2)	−0.010 (3)	0.015 (2)	0.004 (2)
O5	0.064 (2)	0.164 (5)	0.117 (4)	−0.025 (3)	0.002 (3)	−0.039 (4)
O6	0.081 (3)	0.091 (3)	0.096 (3)	0.032 (3)	0.026 (2)	−0.004 (3)
N1	0.052 (3)	0.070 (3)	0.076 (3)	0.001 (3)	0.015 (2)	−0.002 (3)
N2	0.057 (3)	0.074 (3)	0.057 (3)	0.002 (3)	0.004 (2)	0.004 (3)
C1	0.109 (5)	0.110 (6)	0.073 (5)	0.025 (5)	0.010 (4)	0.006 (4)
C2	0.060 (3)	0.048 (3)	0.058 (3)	0.005 (3)	0.014 (3)	0.007 (3)
C3	0.062 (4)	0.051 (4)	0.062 (4)	−0.004 (3)	0.026 (3)	0.010 (3)
C4	0.065 (3)	0.074 (4)	0.074 (4)	0.011 (3)	0.008 (3)	0.015 (3)
C5	0.047 (3)	0.049 (3)	0.066 (4)	0.009 (3)	0.012 (3)	−0.002 (3)
C6	0.038 (2)	0.051 (3)	0.058 (3)	0.000 (2)	0.009 (2)	−0.002 (2)
C7	0.040 (3)	0.048 (3)	0.081 (4)	0.009 (2)	0.023 (3)	0.009 (3)
C8	0.064 (3)	0.056 (4)	0.073 (4)	0.013 (3)	0.031 (3)	0.002 (3)
C9	0.046 (3)	0.073 (4)	0.070 (4)	0.002 (3)	0.013 (3)	0.007 (3)
C10	0.059 (3)	0.069 (4)	0.063 (3)	0.002 (3)	0.023 (3)	0.004 (3)
C11	0.079 (4)	0.108 (6)	0.066 (4)	−0.019 (4)	0.016 (3)	0.001 (4)
C12	0.121 (6)	0.080 (5)	0.053 (4)	−0.014 (4)	0.006 (4)	−0.006 (4)
C13	0.048 (3)	0.048 (4)	0.071 (4)	0.005 (3)	0.013 (3)	−0.005 (3)
C14	0.056 (3)	0.052 (3)	0.056 (3)	0.000 (3)	0.013 (3)	0.004 (3)
C15	0.057 (3)	0.081 (4)	0.063 (4)	0.004 (3)	0.000 (3)	−0.007 (3)
C16	0.056 (3)	0.061 (4)	0.066 (3)	−0.002 (3)	0.028 (3)	0.004 (3)
C17	0.044 (3)	0.053 (3)	0.057 (3)	0.002 (3)	0.011 (2)	0.000 (3)
C18	0.064 (3)	0.051 (3)	0.061 (3)	−0.003 (3)	0.012 (3)	0.006 (3)
C19	0.058 (3)	0.057 (4)	0.070 (4)	−0.007 (3)	0.012 (3)	0.009 (3)
C20	0.043 (2)	0.079 (4)	0.065 (3)	−0.004 (3)	0.016 (2)	0.000 (3)
C21	0.052 (3)	0.066 (4)	0.068 (3)	0.004 (3)	0.021 (2)	0.002 (3)
C22	0.095 (4)	0.138 (7)	0.060 (4)	0.028 (5)	0.028 (3)	0.005 (4)

### Geometric parameters (Å, °)

**Table:**

N1—O2	1.197 (6)	C11—H11A	0.9600
N1—O3	1.221 (6)	C11—H11B	0.9600
N1—C10	1.476 (7)	C11—H11C	0.9600
N2—O5	1.194 (6)	C12—O4	1.452 (8)
N2—O6	1.211 (6)	C12—H12A	0.9700
N2—C21	1.475 (7)	C12—H12B	0.9700
C1—O1	1.446 (8)	C13—C16	1.363 (7)
C1—H1A	0.9700	C13—C14	1.383 (7)
C1—H1B	0.9700	C14—O4	1.363 (6)
C2—C5	1.375 (7)	C14—C19	1.368 (7)
C2—C3	1.386 (7)	C15—C12	1.521 (8)
C3—O1	1.362 (6)	C15—C13	1.521 (7)
C3—C8	1.362 (7)	C15—H15A	0.9700
C4—C1	1.505 (9)	C15—H15B	0.9700
C4—C2	1.506 (7)	C16—C17	1.389 (7)
C4—H4A	0.9700	C16—H16A	0.9300
C4—H4B	0.9700	C17—C18	1.383 (7)
C5—C6	1.395 (7)	C17—C20	1.458 (7)
C5—H5A	0.9300	C18—C19	1.379 (7)
C6—C7	1.404 (6)	C18—H18A	0.9300
C6—C9	1.468 (7)	C19—H19A	0.9300
C7—C8	1.389 (7)	C20—C21	1.331 (7)
C7—H7A	0.9300	C20—H20A	0.9300
C8—H8A	0.9300	C21—C22	1.489 (8)
C9—C10	1.325 (7)	C22—H22A	0.9600
C9—H9A	0.9300	C22—H22B	0.9600
C10—C11	1.490 (7)	C22—H22C	0.9600
			
C3—O1—C1	106.5 (4)	H11A—C11—H11B	109.5
C14—O4—C12	107.4 (4)	C10—C11—H11C	109.5
O2—N1—O3	122.7 (5)	H11A—C11—H11C	109.5
O2—N1—C10	117.9 (5)	H11B—C11—H11C	109.5
O3—N1—C10	119.4 (5)	O4—C12—C15	108.3 (5)
O5—N2—O6	124.5 (5)	O4—C12—H12A	110.0
O5—N2—C21	115.5 (5)	C15—C12—H12A	110.0
O6—N2—C21	120.0 (5)	O4—C12—H12B	110.0
O1—C1—C4	109.0 (5)	C15—C12—H12B	110.0
O1—C1—H1A	109.9	H12A—C12—H12B	108.4
C4—C1—H1A	109.9	C16—C13—C14	118.3 (5)
O1—C1—H1B	109.9	C16—C13—C15	133.8 (5)
C4—C1—H1B	109.9	C14—C13—C15	107.9 (5)
H1A—C1—H1B	108.3	O4—C14—C19	122.9 (5)
C5—C2—C3	119.6 (5)	O4—C14—C13	113.9 (5)
C5—C2—C4	132.0 (5)	C19—C14—C13	123.1 (5)
C3—C2—C4	108.4 (5)	C12—C15—C13	101.8 (5)
O1—C3—C8	124.4 (5)	C12—C15—H15A	111.4
O1—C3—C2	113.2 (5)	C13—C15—H15A	111.4
C8—C3—C2	122.4 (5)	C12—C15—H15B	111.4
C1—C4—C2	101.3 (5)	C13—C15—H15B	111.4
C1—C4—H4A	111.5	H15A—C15—H15B	109.3
C2—C4—H4A	111.5	C13—C16—C17	121.9 (5)
C1—C4—H4B	111.5	C13—C16—H16A	119.1
C2—C4—H4B	111.5	C17—C16—H16A	119.1
H4A—C4—H4B	109.3	C18—C17—C16	116.6 (5)
C2—C5—C6	119.4 (5)	C18—C17—C20	124.1 (5)
C2—C5—H5A	120.3	C16—C17—C20	119.1 (4)
C6—C5—H5A	120.3	C19—C18—C17	124.0 (5)
C5—C6—C7	119.9 (5)	C19—C18—H18A	118.0
C5—C6—C9	117.8 (4)	C17—C18—H18A	118.0
C7—C6—C9	122.2 (5)	C14—C19—C18	116.0 (5)
C8—C7—C6	120.1 (5)	C14—C19—H19A	122.0
C8—C7—H7A	120.0	C18—C19—H19A	122.0
C6—C7—H7A	120.0	C21—C20—C17	132.5 (5)
C3—C8—C7	118.7 (5)	C21—C20—H20A	113.8
C3—C8—H8A	120.7	C17—C20—H20A	113.8
C7—C8—H8A	120.7	C20—C21—N2	118.3 (5)
C10—C9—C6	132.0 (5)	C20—C21—C22	127.9 (5)
C10—C9—H9A	114.0	N2—C21—C22	113.7 (5)
C6—C9—H9A	114.0	C21—C22—H22A	109.5
C9—C10—N1	121.4 (5)	C21—C22—H22B	109.5
C9—C10—C11	126.3 (6)	H22A—C22—H22B	109.5
N1—C10—C11	112.4 (5)	C21—C22—H22C	109.5
C10—C11—H11A	109.5	H22A—C22—H22C	109.5
C10—C11—H11B	109.5	H22B—C22—H22C	109.5
			
C2—C3—O1—C1	7.9 (7)	C7—C6—C9—C10	−30.1 (10)
C4—C1—O1—C3	−12.6 (7)	C6—C7—C8—C3	0.3 (8)
C8—C3—O1—C1	−172.9 (6)	C6—C9—C10—N1	−5.0 (11)
O2—N1—C10—C9	129.9 (6)	C6—C9—C10—C11	174.6 (6)
O3—N1—C10—C9	−49.9 (8)	C15—C12—O4—C14	7.6 (7)
O2—N1—C10—C11	−49.8 (7)	C15—C13—C14—O4	−2.1 (6)
O3—N1—C10—C11	130.4 (6)	C15—C13—C14—C19	−178.9 (5)
O5—N2—C21—C20	−131.9 (6)	C16—C13—C14—O4	177.5 (5)
O5—N2—C21—C22	45.6 (8)	C16—C13—C14—C19	0.7 (8)
O6—N2—C21—C20	50.6 (8)	C14—C13—C16—C17	1.2 (8)
O6—N2—C21—C22	−132.0 (6)	C15—C13—C16—C17	−179.4 (6)
C5—C2—C3—O1	−179.7 (5)	C19—C14—O4—C12	173.4 (6)
C4—C2—C3—O1	−0.1 (7)	C13—C14—O4—C12	−3.5 (6)
C5—C2—C3—C8	1.0 (8)	O4—C14—C19—C18	−177.4 (5)
C4—C2—C3—C8	−179.3 (6)	C13—C14—C19—C18	−0.8 (8)
C3—C2—C5—C6	−0.8 (8)	C13—C15—C12—O4	−8.3 (7)
C4—C2—C5—C6	179.7 (6)	C12—C15—C13—C14	6.3 (6)
O1—C3—C8—C7	−180.0 (5)	C12—C15—C13—C16	−173.2 (6)
C2—C3—C8—C7	−0.8 (8)	C13—C16—C17—C18	−2.7 (8)
C2—C4—C1—O1	12.0 (7)	C13—C16—C17—C20	−177.7 (5)
C1—C4—C2—C5	172.3 (6)	C16—C17—C18—C19	2.6 (8)
C1—C4—C2—C3	−7.3 (7)	C20—C17—C18—C19	177.4 (5)
C2—C5—C6—C7	0.4 (8)	C18—C17—C20—C21	32.8 (10)
C2—C5—C6—C9	176.9 (5)	C16—C17—C20—C21	−152.5 (7)
C5—C6—C7—C8	−0.1 (8)	C17—C18—C19—C14	−0.9 (8)
C9—C6—C7—C8	−176.5 (5)	C17—C20—C21—N2	6.0 (10)
C5—C6—C9—C10	153.4 (7)	C17—C20—C21—C22	−171.0 (7)

### Hydrogen-bond geometry (Å, °)

**Table:**

*D*—H···*A*	*D*—H	H···*A*	*D*···*A*	*D*—H···*A*
C7—H7A···N1	0.93	2.58	3.096 (8)	116
C18—H18A···N2	0.93	2.60	3.094 (7)	114
C4—H4A···Cg4	0.97	2.88	3.673 (8)	140
C8—H8A···Cg3^i^	0.93	2.92	3.578 (8)	129
C15—H15B···Cg2	0.97	2.85	3.636 (7)	139
C19—H19A···Cg2^ii^	0.93	2.75	3.497 (7)	139

Symmetry codes:  (i) −*x*+1, *y*+1/2, −*z*+1; (ii) −*x*+2, *y*−1/2, −*z*+1.

## References

[bb1] Allen, F. H., Kennard, O., Watson, D. G., Brammer, L., Orpen, A. G. & Taylor, R. (1987). *J. Chem. Soc. Perkin Trans. 2*, pp. S1–19.

[bb2] Enraf–Nonius (1989). *CAD-4 Software* Enraf–Nonius, Delft, The Netherlands.

[bb3] Farrugia, L. J. (1997). *J. Appl. Cryst.* **30**, 565.

[bb4] Harms, K. & Wocadlo, S. (1995). *XCAD4* University of Marburg, Germany.

[bb5] North, A. C. T., Phillips, D. C. & Mathews, F. S. (1968). *Acta Cryst.* A**24**, 351–359.

[bb6] Sheldrick, G. M. (2008). *Acta Cryst.* A**64**, 112–122. 10.1107/S0108767307043930 18156677

[bb7] Spek, A. L. (2009). *Acta Cryst.* D**65**, 148–155. 10.1107/S090744490804362X PMC2631630 19171970

